# Phytoradiotherapy: An Integrative Approach to Cancer Treatment by Combining Radiotherapy With Phytomedicines

**DOI:** 10.3389/fonc.2020.624663

**Published:** 2021-02-08

**Authors:** Tyler Alfonzetti, Sayeda Yasmin-Karim, Wilfred Ngwa, Stephen Avery

**Affiliations:** ^1^ Department of Radiation Oncology, Perelman Center for Advanced Medicine, University of Pennsylvania, Philadelphia, PA, United States; ^2^ Department of Radiation Oncology, Dana Farber Cancer Institute, Brigham and Women’s Hospital, Harvard Medical School, Boston, MA, United States

**Keywords:** phytomedicine, radiotherapy, phytoradiotherapy, cannabinoids, bitter melon

## Abstract

Radiotherapy (RT) is an effective method of cancer treatment, but like any other method of cancer treatment, there are inherent limitations. While technological advances and a growing understanding of its biological effects have improved its results dramatically, the use of RT is still limited to certain patient populations and by normal tissue toxicities. The harmful side effects of treating patients with radiation can offset its therapy benefits, limiting its use in certain cases. Phyto, or plant-based, medicines offer a way to add to radiation treatment, while also protecting patients from its toxic side effects. Phytomedicines such as cannabinoids (CBD) and bitter melon extract have demonstrated therapeutic properties, including the ability to activate apoptotic death in cancer cells, diminish tumor progression, and generally decrease the incidence of several cancer types. In addition, herbal drugs have been shown to be powerful antioxidants with the ability to decrease toxicity of RT without the adverse side effects found in synthetic drugs. Furthermore, a number of phytomedicines have been shown to mitigate hypoxic conditions within the tumor microenvironment, creating a more radiosensitive disease and preventing tumorigenesis. The purpose of this article is to examine the merits and demerits of employing phytomedicines during RT. Results from studies that have tested the effects of combining radiotherapy with supplemental herbal treatment are discussed along with perspectives on where additional research is needed to advance “Phytoradiotherapy”. Overall, experimental evidence points to the fact that phytomedicines have significant potential to enhance RT, with need for cross-disciplinary collaborations to establish optimal dosing combinations with evidence-base for clinical translation.

## Introduction

Phytoradiotherapy is the integration of radiotherapy (RT) with phyto- or plant-based medicines to enhance cancer treatment. Phytomedicines have potential to not only enhance the therapeutic effects of radiotherapy but mitigate the damaging side effects as well.

Phytomedicines are plant-based pharmaceuticals or extracts used to treat different ailments. 2019 data taken from the World Heath Organization (1) shows over 60% of people around the globe rely on natural herbs to provide themselves and their families with healthcare. With an increasing number of publications supporting the effectiveness of phytomedicines in oncology and their antioxidant potential, it is hypothesized that combining them with radiotherapy can significantly impact the overall survival of cancer patients, and their quality of life. This constitutes the cross disciplinary area of Phytoradiotherapy, combining radiotherapy with phyto- or plant-based medicines to enhance cancer treatment and/or minimize toxicities.

Conservative estimates from recent years indicate that more than 50% of cancer patients can benefit from radiation therapy (2) which has been playing an increasingly important role in cancer treatment. In spite of its effectiveness and widespread adoption, radiation therapy use for curative treatment of certain cancers is limited. This is largely due to cancers not being found until they are late stage, as well complex patient heterogeneity. In addition, radiation therapy can lead to secondary cancers later in a patient’s life as well as cause harmful side effects during and after treatment.

In this article, we first examine the merits of using phytomedicines such as cannabinoids in oncology. This is followed by a review of work in the emerging area of Phytoradiotherapy, with perspectives on the promise and limitations of this approach. Future directions and perspective on key areas for research to justify clinical translation and adoption are discussed.

## Phytomedicine

Phytomedicines, medicinal plants for prevention and treatment of disease, are of growing importance worldwide (3). For so many of the 4 billion across the world who use phytomedicine, it is their mainstay of health care delivery, and demand for it is increasing. Phytomedicine of proven quality, safety, and efficacy, contributes to the World Health Organization’s global health priority of ensuring that all people have access to quality healthcare. However, the use of phytomedicines in cancer treatment is largely driven by anecdotal evidence (4), and is limited by poor bioavailability. Nature has, in fact, been making biologically active compounds in plants for billions of years. Examples of medicines from plants that have a major impact globally include: morphine from opium Poppy (Papaver somniferum), aspirin from the white willow tree (Salix alba vulgaris), and the anticoagulant coumadin from spoiled sweet clover (Melilotus species). Tropical plants such as the Madagascar Periwinkle (Catharanthus roseus) have yielded vinblastine, which has revolutionized the treatment of Hodgkin’s lymphoma, and vincristine, which has done same for acute childhood leukemia.

Over 33 states in the USA, Canada, and a growing number of countries have now legalized medical cannabis, with studies showing promising approaches for use of cannabinoid and flavonoid components of medical Cannabis in oncology (4, 5). The National Institutes of Health, and World Health Organization encourage research on medical cannabis for treating different diseases including cancer, cancer pain, and managing the side effects of other treatments such as radiotherapy or chemotherapy. Besides Cannabis, it is estimated that of the 300,000 plant species that exist in the world, only 15% have been evaluated to determine their pharmacological potential.

While some studies have not demonstrated plant-based herbs as effective forms of medicine, especially in oncology (6, 7), there are a plethora of studies, that support phytomedicines as a valuable form of medicine. With many reported studies on natural herbs use in the treatment of cancer and other disease, there is impetus for more research in this area. In addition, the delivery method of phytomedicines may determine their efficacy. Often, modes of phytomedicine delivery like oral administration or intravenous injection may limit their efficacy, even if they demonstrate efficacy in-vitro. Recent advances in technology have led to the development of smart biomaterials that can enable targeted controlled delivery of phytomedicines to their intended targets with sustained bioavailability (8). Experimentations with these materials show their ability to dramatically improve the effects of their phytomedicine payloads. For example, in-vivo studies in mice have shown that smart biomaterials can be developed to administer cannabinoids in combination with radiation therapy for enhanced therapeutic efficacy (9).

Phytomedicines also have palliative applications as in managing pain and have the benefit of being accessible and less expensive compared to other therapeutics as opioids. Cancer treatment can be a very costly endeavor introducing huge amounts of financial pressure on patients. At the University of Pennsylvania, photon intensity modulated radiotherapy (IMRT) and Proton Therapy treatments can cost approximately 20,000 and 30,000 dollars, respectively. It was cited that certain institutions report the cost of their IMRT treatment plans as costing up to over 100,000 US dollars (10). Besides radiotherapy, drugs given to cancer patients are accompanied by an overwhelming price tag. For example, the drug ipilimumab was approved in 2015 by the FDA to help treat metastatic melanoma after it showed the ability to prolong survival of patients by an average of approximately 1.6 months (10). Just four doses of the drug cost 120,000 dollars. Unfortunately, this is not an anomaly; Cancer drugs including Sipuleucel-T, Bevacizumab, and more cost over 80,000 dollars for just a few doses (11). The high cost of such drugs is just one reason the economic burden of cancer in the US is expected to increase substantially in the future. Coupled with the growing population and upward trends in costs of diagnosis/care call attention to finding ways that can help reduce expenses (12). Overall, the tremendous cost of modern-day cancer treatment can be a difficult obstacle to overcome, especially for patients in low/middle income (LMIC) countries. With life expectancy and world population rising, the cancer burden throughout the globe is growing. In many LMICs, phytomedicines constitute a major part of the healthcare system, and it is worth considering evidence-based cost-effective approaches that integrate phytomedicines, which may enhance therapeutic efficacy or enhance safety.

Data reported in the National Business Journal (13) show the growing market in the United States for herbal supplements. From an economic perspective, there may also be benefits for increased adoption of evidence-based phytomedicines in oncology in the USA. The economic impact of phytomedicines is three-fold: reducing the cost of cancer treatment, providing affordable care for patients in developing countries, and boosting the economies of both developing and developed nations.

In a paper reviewing the cost effectiveness of adding phytomedicine to chemotherapy, the cost per annum of phytomedicine per patient was estimated to about $1,000 in India (14). According to Cancer.gov most cancer drugs launched between 2009 and 2014 cost over $100,000 per patient for one year of treatment. It is important to note that the quote of $1,000 for a year’s worth of phytomedicine can be misleading. These two prices are comparing drugs that do not have identical effects and are not used in identical cases therefore, the discrepancy in these two prices is less significant. However, the clear conclusion is that phytomedicines are substantially less costly to patients than synthetic drugs.

Prevalent poverty and limited resources make it difficult to protect the health of citizens (12). Interestingly, many of the plants desirable to be used in medication are found in places near developing countries. The harvesting and sale of such plants delivers economic opportunity. Global statistics indicate that the importance of medicinal plants or plant-based pharmaceuticals is continuously growing (15). For example, in Pakistani communities many citizens often earn additional income by collecting and selling plants used in herbal medicine (16). Further development of phytomedicines as an integral part of cancer treatment will help emphasize these markets in areas like Pakistan. Developing countries can harness these markets and export Phytopharmaceuticals improving their healthcare system and economy. Rwanda, and Uganda are two examples in Africa that have recently approved growth and export of Medical Cannabis. The primary benefit of phytomedicines, however, will be its effectiveness in the clinic.

## Phytoradiotherapy

Combining RT and phytomedicines will have the greatest potential impact in improving cancer treatment by (1) enhancing the therapeutic efficacy of RT and (2) minimizing the harmful side effects associated with RT.

### Enhancing Therapeutic Efficacy

**Figure 1:**
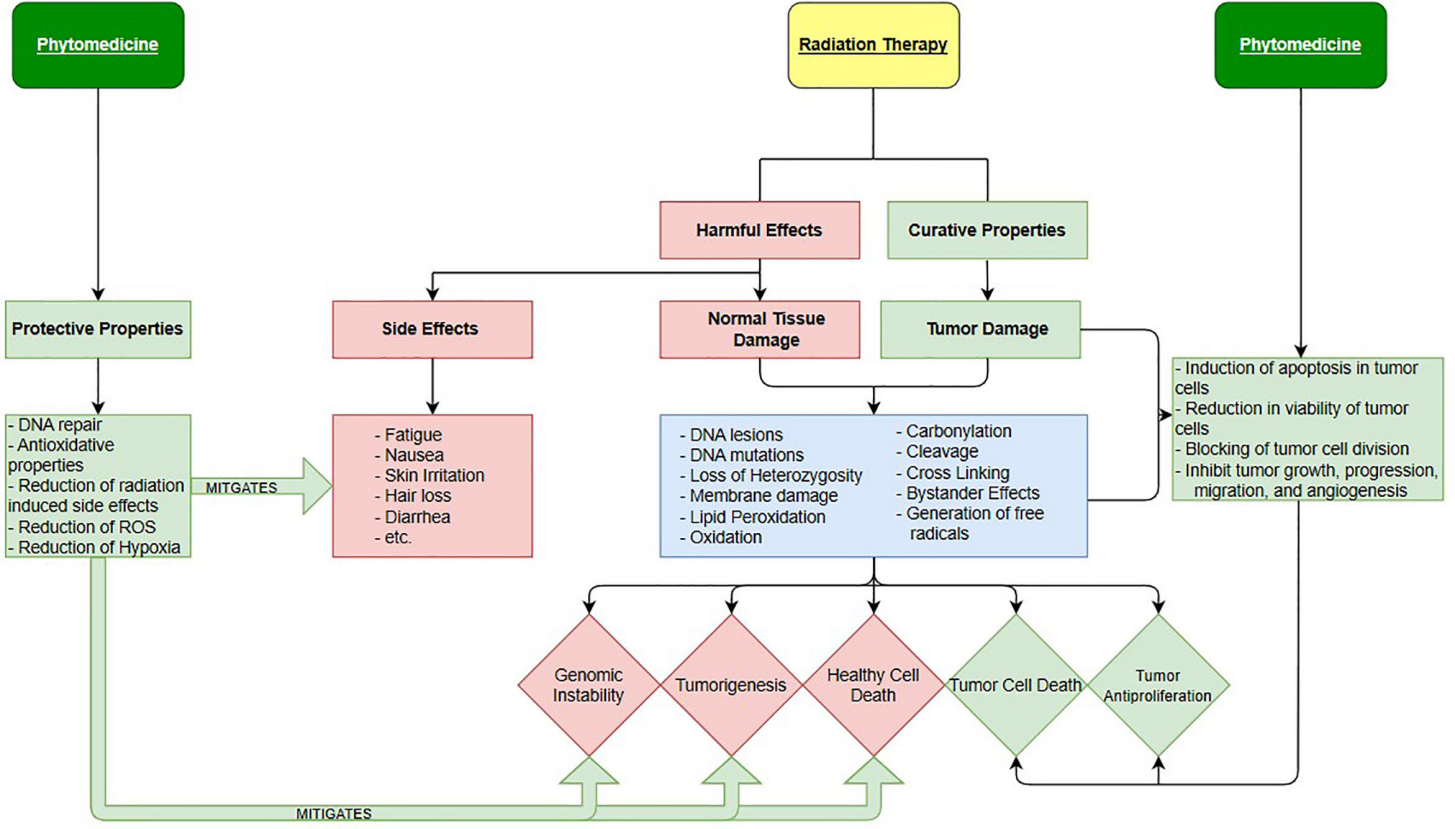
Harmful and Curative Properties of Radiation Therapy and Phytomedicines.

In human cancers, a deficiency in the amount of oxygen reaching tissues, or hypoxia, is common and leads to an increase probability of mortality. Under these conditions, Hypoxia inducible factors (HIF) mediate the body’s physiological response (17). In tumors, HIF transcription is heightened so that cancer cells can survive in hypoxic conditions where healthy cells would have normally died. In addition, in environments lacking oxygen, ionizing radiation is far less lethal, meaning that radiotherapy is less effective. Hypoxia is one of the most important causes of radiotherapy failure due to the hypoxic cells being immune to the reactive oxygen species created by incident ionizing radiation (18, 19). Anemia is a condition in which the victim lacks a sufficient number of healthy red blood cells to adequately supply the body with oxygen (20–23). Since anemia brings on hypoxia-like conditions and it is often a symptom of cancer it presents a serious problem for those who rely on radiation therapy for a cure. There is growing evidence describing how phytomedicines can help reduce the hypoxic resistance of cancer cells by reducing the transcription of the Hypoxia-Inducible factors. This could explain why anti-cancerogenic phytomedicines like Cannabidiol (24–28) displayed synergistic effects against cancer cells *in vitro*. Studies showing the anti-cancer effects of cannabinoids (29) and their ability to manage cancer related symptoms (25, 29) are plentiful. In fact, there are even a few isolated studies, showing the effectiveness of using cannabinoids (CBD) and other phytomedicines with radiation therapy together. In addition to CBD, some other specific plant-based medicines with therapeutic potential include Bitter Melon Extract, *Picrorhiza kurrooa*, Justica (20), and many more. Schematic overview of integration of radiation therapy and phytomedicines is shown in **Figure 1**.

### Minimizing the Harmful Effects of Radiotherapy (Antioxidant Properties)

Ionizing radiation mostly causes direct cell killing by damaging the cell’s DNA. Once the DNA is damaged with double-strand breaks beyond correct repair, the cell loses its function and dies (30). Naturally, the aim of radiotherapy is to use ionizing radiation to kill tumor cells while avoiding any healthy cells. Such a goal is a lot easier said than done as the effects of ionizing radiation are dependent on several factors (31). Cells can also be killed indirectly by ionizing radiation due to hydroxyl free radicals which combine with other substances that lead to cell, tissue, and organ damage. The excess of these harmful free radicals created in the target areas of radiation therapy produce conditions that are medically described as “oxidative stress”, causing the onset of several different diseases. Of course, tumor cell death is a positive outcome, but in healthy tissue/organs the oxidative damage they cause can be detrimental (32). The DNA damage caused by oxidative stress can mutate genes leading to the development of cancer (33). These genes are known as Oncogenes and are often described as cancer stem cells.

Oncologists must consider a balance between the reduction/production of Reactive Oxygen Species (ROS) so that tumor cell killing is promoted while dangerous side effects are avoided. Substances with antioxidative properties can help control this balancing act. Many synthetic drugs have been developed to mitigate the toxic effects of these free radicals but introduce health problems of their own. One study from 1993 tested synthetic antioxidants Butylated hydroxyanisole (BHA) and Butylated hydroxytoluene (BHT) and found that they induce both blood clotting impairment and certain types of tumors in animal models. In contrast, the study showed no carcinogenic properties performing the same studies with Vitamin E being used as a natural antioxidant (34).

We see, in **Figure 1**, for radiotherapy the harmful effects and curative properties are one and the same, both involving the damaging of cells by the same mechanisms. The difference between the two is whether the ionizing radiation interacts with healthy or cancerous cells. However, normal tissues in the human body generally exhibit superior cell repair compared to cancerous cells, attributing to the modern success of radiation therapy. Besides DNA damage, carbonylation, cleavage, loss of Heterozygosity and other events also cause damage, if not death (35). The killing of healthy cells causes the three biological outcomes seen as red diamonds while killing of cancerous cells creates auspicious results represented as the two green diamonds. Notice that the effects of phytomedicines (when used correctly) are only colored green indicating that they only introduce positive effects into cancer treatment. This is not to imply that phytomedicine are never toxic. Certain phytomedicines can be very harmful and even medicinal ones can have toxic effects if a wrong preparation or dose is used. On the right side, we see phytomedicines therapeutic properties at work enhancing tumor cell damage while not contributing to any significant healthy cell death. On the left, the radio-protective properties of phytomedicines are displayed. The protective properties that plant-based pharmaceuticals possess act to mitigate toxic side effects of radiation therapy while also limiting healthy cell death.

In 2016, a study on Plant Derived Antioxidants in Disease Prevention (36) showed that phytomedicines act successfully as suppressors of oxidation and suggested they should be used in the future as medicinal agents. Extracts from several plants including Green Tea (6, 37–39), Bitter Melon (40, 41) and more display remarkable therapeutic properties. Extracts from these plants were also seen to inhibit growth of malignant melanoma cancers and kill Leukemia cells. The anti-tumorigenesis properties of plants like these would not only enhance a patient’s radiation therapy directly by promoting tumor control, but also minimize side effects, with their antioxidative properties, that are often a contributing factor to an unsuccessful radiotherapy treatment. **Table 1** organizes a fraction of the evidence from reviewed literature on using phytomedicines to treat a variety of cancer sites. From the information in this table, we can see a variety of phytomedicines used to treat different sites. For example, *Vincea rosea* and *Pargaum harmala L.* were used effectively against cervical cancers while herbs such as Curcumin and *Stephania Tetranda* were used to battle brain cancers.

**Table 1:** Anticancer Properties of Phytomedicines for the Treatment of Leukemia, Pancreatic, Lung, Abdominal, Brain, Cervical and Breast Cancers.

	Phytomedicine	Description	Result	Supplemental Treatment	Ref.
**Leukemia**	*Ligusticum* *Wallichii*	Leukemia cell line U937 cells were treated with different concentration of Tetramethylpyrazine	Tetramethylpyrazine significantly inhibited the cell growth of Leukemia cells	none	(42)
	*Cannabidiol* *(CBD)*	Examined effects of cannabidiol on induction of apoptosis in leukemia cells	Reduction in cell viability and induction of apoptosis. Significant decrease in tumor burden and increase in apoptotic tumors in vivo	none	(29)
	*Salix safsaf* *(Willow Leaves)*	Test extract from *Salix safsaf* for activity against human carcinoma cells in vitro and in vivo	Killed large portion of tumor cells with minimal effects on normal cells Prolonged Survival/inhibited tumor growth in animals treated with willow leaf extract	none	(43)
**Lung Cancer**	*Rhus Verniciflua* *(Lacquer Tree)*	Assess the prolongation of survival in non-small cell lung cancer (NSCLC) patients	The median survival time and survival rate increased in patients who were treated with *Rhus Verniciflua*	Chemotherapy	(44)
	*Brucea javanica*	Patients with brain metastasis as a complication of lung cancer were either treated with radiotherapy alone or in combination with *Brucea javanica*	Patients receiving the Brucea javanica treatment showed a 5-month increase in median survival compared to those treated with radiation therapy alone Quality of life was reported to be much better in the treated group than the control group (radiotherapy alone)	Radiotherapy	(45)
	*Cannabidiol* *(CBD)*	Test the effects of combining radiotherapy and CBD oil treatment on lung cancer in vitro an in vivo	In vitro, substantially enhanced tumor cell killing was seen when using CBDs with radiation therapy In vivo, mice treated with CBD and radiation showed the least amount of tumor growth and longest greatest survival percentage	none	(9)
**Pancreatic Cancer**	*Cannabidiol* *(CBD)*	Test the effects of combining radiotherapy and CBD oil treatment on lung cancer in vitro and in vivo	In vitro, substantially enhanced tumor cell killing was seen when using CBDs with radiation therapy In vivo, mice treated with CBD and radiation showed the least amount of tumor growth and longest greatest survival percentage	Radiotherapy	(9)
	*Bitter Melon Extract* *(BME)*	Assess the novel agent bitter melon juice against pancreatic carcinoma cell both in culture and nude mice	BMJ decreased cell viability in all four pancreatic carcinoma cell lines by inducing strong apoptotic death In vivo orally administered BMJ prohibited pancreatic tumor growth by 60% with no noticeable toxicity	none	(40)
	*Oplopanax horridus* *(Devils Club)*	Comparing antiproliferation activity of chemotherapy drugs alone and with Devils Club (DC) extract on human pancreatic cancer multicellular spheroids	DC extract significantly enhanced the antiproliferation activity of gemcitabine and cisplatin (two common chemotherapy drugs)	Chemotherapy	(46)
**Brain Cancer**	*Stephania* *tetrandra*	Test the radiosensitization effects of Tetrandrine which can be extracted from Stephania tetrandra on human glioma using flow cytometric analysis and investigate expression of phosphorylated H2AX	Confirmed Tetrandrine as a radiosensitizer on human glioma through inhibiting proliferation survival and angiogenesis of glioma cells	Radiotherapy	(47)
	*Quercetin*	Investigate the inhibitory effects of the chemo preventive flavonoid quercetin on glioblastoma cells	Exposure to Quercetin resulted in a marked decrease of proliferative and migratory properties of glioblastoma cells	None	(48)
	*Curcumin*	Experiment with the anticancer properties of Curcumin against glioblastoma cells	Curcumin was found to inhibit tumor cell growth, induce tumor cell cycle arrest, and inhibit metastases. It was also found that Curcumin is a radiosensitizer	Radiotherapy	(49)
**Abdominal Cancer**	*Green Tea*	Test the hypothesis that green tee consumption reduces cancer risk by performing large population-based case-control study	Increasing amount of green tea consumption resulted in lesser association with cancers of the colon, rectum, and pancreas	none	(38)
	*Cannabidiol* *(CBD)*	Tested pure cannabinoids and extracts *Cannabis* strains on Androgen Receptor (AR) positive and negative human prostate adenocarcinoma cells	CBD significantly inhibited cell viability, induced apoptosis, and induced intrinsic apoptotic pathways	none	(50)
	*Boswellia serrata*	Assess anti-cancer effect of Boswellia serrata extract on HeLa cell lines using MTT assay (testing Succinate dehydrogenase activity to determine cell viability)	*Boswellia serrata* has dose dependent and time dependent anti-cancer effects on HeLa cells by inhibiting their growth	none	(51)
**Breast Cancer**	*Bitter Melon Extract* *(BME)*	Test antiproliferative effects in detail of Bitter Melon Extract on breast cancer cells in animals	BME inhibits breast tumor growth by, in part, inducing autophagy and apoptotic cell death in animals	none	(41)
	*Withania somnifera*	Recorded the fatigue and median survival of breast cancer patients receiving chemotherapy alone vs. chemotherapy in conjunction with *Withania* somnifera treatments	Reduced Fatigue in patients receiving *Withania somnifera*	Chemotherapy	(52)
	*Standardized Mistletoe Extract*	Investigated effects of standardized mistletoe extract reported significantly fewer disease/therapy related symptoms and improved quality of life than those that did not receive the additional treatment.	Patients receiving standardized mistletoe extract reported significantly fewer disease/therapy related symptoms and improved quality of life than those that did not receive the additional treatment	Surgery Chemotherapy Radiotherapy Hormone Therapy	(53)
**Cervical Cancer**	*Pegaum harmala L*	Investigate the effective medicinal plants in the treatment of cancer and study their mechanisms of action	The extract of *Pegaum harmala L* reduced the viability of epithelial cervical carcinoma cells	none	(51)
	*Rosa damascena Extract*	Uncover chemotherapeutic properties of *Rosa damascene* extract against HeLa cells	*Rosa damascene* extract decreased cell viability in malignant cells in a concentration and time dependent manner *Rosa damascena* could be considered a promising chemotherapeutic agent in future cancer treatment	none	(54)
	*Vincea rosea*	Test the effects of the plant’s Alkaloids in HeLa cancer cells	The Alkaloids present in *Vincea rosea* blocked the division of cancerous cells and its antioxidant properties prevented cancerous cells from progressing	none	(55)

## Discussion and Future Perspective

Natural herbs and plants have been an integral part of medicine throughout all human history and despite a relatively recent shift towards more western medicines, many areas of the world still rely heavily on these phytomedicines. The World Health Organization has estimated that 80% of the world population uses herbal or complementary medicine (56) and scientific evidence has already defended the hypothesis that combinations of traditional and western medicines improve prognosis of deadly cancers (57). Upon reviewing a plethora of experimental evidence showing a wide variety of phytomedicines being used to successfully treat different types of cancers, there is overwhelming support for their increased use as an effective part of treatment. This is why organizations like the National Institute of Health support further research on traditional medicines and integrative health practices (58). The organization has placed emphasis on finding ways to curb the crippling opioid problem that is currently devasting the United States of America (59) as well as enhancing pain management for patients suffering from agonizing ailments. Emerging from this line of thought is a promising way to revolutionize radiation therapy to manage a large portion of the world’s cancer. Phytomedicines have potential to not only effectively reduce side-effects and symptoms of cancer but boost the treatment itself.

The pursuit of Phytoradiotherapy is limited by the lack of clinical trials that integrate the two treatments. There is an overwhelming amount of literature discussing phytomedicines and radiotherapy separately, but not nearly enough has been done experimenting with the two used in combination. In addition, a deeper economic analysis should be employed before drawing conclusions about stimulating economic growth that is more quantitative rather than theoretical. More work in this regard, especially cross-disciplinary collaborations are required to draw evidence-based conclusions that will move Phytoradiotherapy forward.

## Data Availability Statement

The original contributions presented in the study are included in the article/supplementary material. Further inquiries can be directed to the corresponding author.

## Author Contributions

TA is a student researcher and first author. WN and SA are research mentors and collaborators.WN also contributed funding towards the study of the project. SY-K is a collaborator and has contributed data towards the use of phytomedicines. All authors contributed to the article and approved the submitted version.

## Funding

This work was partially funded by the Department of Radiation Oncology Summer Research Grant at the University of Pennsylvania and the Department of Radiation Oncology at Brigham and Women’s Hospital of Harvard University.

## Conflict of Interest

The authors declare that the research was conducted in the absence of any commercial or financial relationships that could be construed as a potential conflict of interest.
